# Peritoneal inclusion cysts in a young male: A case report

**DOI:** 10.1016/j.ijscr.2023.108248

**Published:** 2023-04-23

**Authors:** Callie Killoran, Danniel Badri, Alexandra Walton, Joanna Perry-Keene, Nicolas Copertino

**Affiliations:** a Department of General Surgery, Sunshine Coast university Hospital, 6 Doherty St, Birtinya, QLD 4575, Australia; b Department of Pathology, Sunshine Coast University Hospital, 6 Doherty St, Birtinya, QLD 4575, Australia

**Keywords:** Peritoneal cyst, Benign, Cystic, Mesothelioma, Debulking surgery, Case report

## Abstract

**Introduction and importance:**

Peritoneal inclusion cyst is a rare benign condition with low potential for malignant transformation but high recurrence rates. Debulking surgery is the recommended first line management for these patients, however, recurrence rates are up to 50 % (Padmanabhan et al., 2020; Chapel and Husain, 2021).

**Case presentation:**

A 26-year-old male being worked up for non-specific abdominal pain with cross-sectional imaging showing multiple multicystic lesions in the abdomen and pelvis. There was a pre-operative suspicion of Pseudomyxoma Peritonei and decision was made for diagnostic laparoscopy and biopsy. Mucin and an abnormal small bowel mesentery was found intraoperatively and sampled leading to the diagnosis of peritoneal inclusion cyst.

**Clinical discussion:**

Treatment of peritoneal inclusion cyst range from surveillance to aggressive treatment with complete cytoreductive surgery with involved field peritonectomy and hyperthermic intra-peritoneal chemotherapy.

**Conclusion:**

First line management of peritoneal inclusion cysts is for debulking surgery. Arguments for less invasive and more aggressive management has been proposed, however, further data needs to be collected to determine gold standard of treatment.

## Highlights

• Peritoneal inclusion cysts previously known as multicystic peritoneal mesothelioma
• Benign pathology but has a high risk of recurrence
• Most commonly affects women over men
• Current mainstay treatment is debulking surgery.

## 1 Introduction

Peritoneal inclusion cyst, otherwise known as multicystic peritoneal mesothelioma (MCPM), is a rare variant of peritoneal mesothelioma [1]. It is a benign condition of uncertain pathogenesis, where multiloculated cysts are formed from reactive mesothelial proliferation [2], [3]. It is most common in women of reproductive age and thought to be due to chronic peritoneal inflammation secondary to endometriosis, pelvic inflammatory disease or previous surgery [1], [2], [4]. Less than 200 cases are reported in the literature with an incidence of 0.15/100,000 annually, thus making the pathology difficult to treat [1], [2], [4], [5]. Peritoneal inclusion cyst has a good prognosis and low malignant potential, but has a recurrence rate up to 50 % [1], [2]. Current first line treatment is debulking surgery, however, given the potential for transformation to invasive mesothelioma and the high recurrence rate, some institutions are treating with more aggressive approaches such as complete cytoreductive (CRS) surgery, peritonectomy and hyperthermic intra-peritoneal chemotherapy (HIPEC) to aim for complete disease resolution [1], [4]. At this stage, there is insufficient data for aggressive treatment to be considered first-line [4]. We present a rare case of peritoneal inclusion cyst in a 26 year old male. This case is reported in line with the SCARE criteria and informed consent was obtained from patient for publication of case report [6].

## 2 Presentation of case

**Fig. 1:**
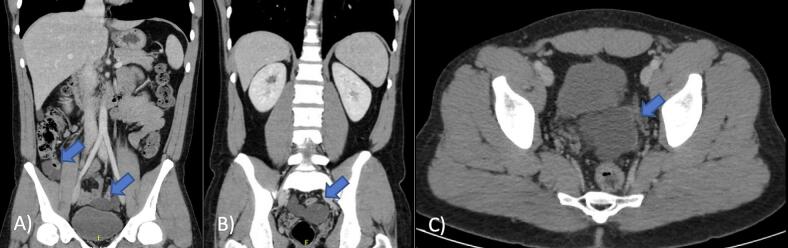
Axial and coronal images of the multicystic lesions in the abdomen. Blue arrow pointing to cystic lesions. A) Lesion near appendix and superior to bladder, B) + C) pelvic cystic lesion between rectum and bladder.

A 26-year-old male, referred by family practitioner, presented with vague lower abdominal pain, bloating, abdominal dysuria and changes to bowel habits for a five-month duration. He reported no history of weight loss, night sweats or changes to appetite. His past medical, drug and social history was unremarkable. He had a benign abdominal examination with no masses palpable. Bloods and tumour markers, colonoscopy, and upper endoscopy revealed no abnormalities. Computerized tomography (CT) of the abdomen and pelvis revealed multiple loculated cystic masses throughout the abdomen and pelvis with a dominant lesion in the pelvis (Fig. 1). Provisional diagnosis of Pseudomyxoma Peritonei (PMP) was made based off imaging appearances. After evaluation and discussion with our local specialist centre for PMP, the decision was to proceed with diagnostic laparoscopy and biopsy.

**Fig. 2:**
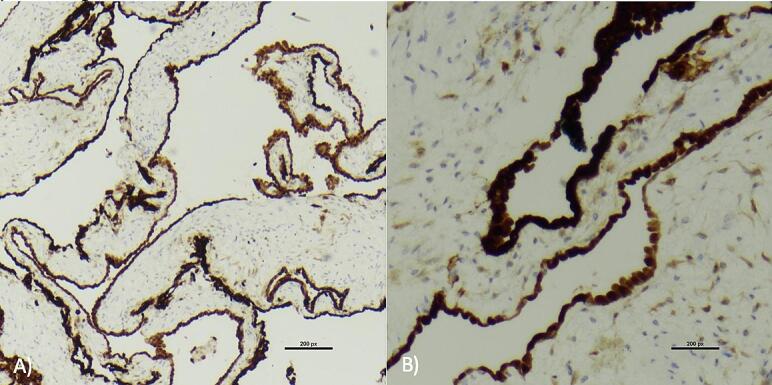
Immunostaining of cells with calretinin. Uptake suggests mesothelial origin. Low (A) and high (B) power magnification.

**Fig. 3:**
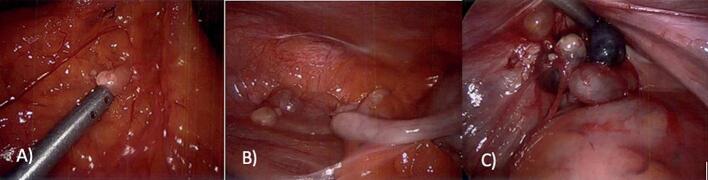
Intraoperative photos from debulking and appendicectomy procedure. A) Omental cyst, B) appendiceal cysts, and C) pelvic cystic mass.

In October 2022, the patient underwent a diagnostic laparoscopy. Free mucin was found along the right side of the abdomen and pelvis, at the tip of the appendix and the small bowel appeared nodular. Pelvic fluid, visible mucin and peritoneal biopsies were taken. Pathological examination of the mucin showed multiloculated cystic spaces lined by flattened to columnar epithelial cells with features favouring mesothelial cells. Immunostaining confirmed the lining cells to be mesothelial with calretinin and WT1 (Fig. 2). No signs of malignancy were seen. It was reported that the gross and microscopic findings were most consistent with a diagnosis of peritoneal inclusion cyst, or MCPM. He recovered well after surgery and postoperative course was uneventful. He was referred onto a tertiary centre for debulking surgery and appendicectomy. At the second surgery, intraoperative findings showed no free mucin but new pelvic cystic mass, cysts on the omentum and multiple cysts at the tip of the appendix (Fig. 3). The patient underwent excision of the cysts, cystic pelvic mass and an appendicectomy. Histo again confirmed peritoneal inclusion cysts at all sites resected. His postoperative course was uneventful and a discussion at the colorectal multidisciplinary team meeting recommended for surgical surveillance without further treatment.

## 3 Discussion

Peritoneal inclusion cysts are extremely rare. They account for 3–5 % of peritoneal mesothelial lesions, however, are benign with a low rate of transformation into malignancy [1], [2]. The tumours occur more frequently in females (80–90 %) and most common in third to fourth decades [2]. The pathology predominantly involves the omental peritoneal surfaces and pelvic visceral surfaces (uterus, fallopian tubes and ovaries) [1]. Diagnosis can be difficult as symptoms are non-specific. Typical patient complaints include diffuse abdominal pain and pressure symptoms. The large intraabdominal cysts can compress pelvic organs and lead to altered bowel and urinary habits. Cross sectional imaging is required for workup and shows intraabdominal, primarily pelvic, multicystic lesions. This can lead to a large differential list in the female pelvis and it is suggested that magnetic resonance imaging (MRI) is helpful in determining origin of the lesion and if there is a solid or liquid component [7]. Differentials during workup include PMP, mucinous cystic neoplasm, lympangiomas, adenomatoid tumours, and in females benign and malignant tumours of the ovaries [1].

Treatment options for confirmed or suspected peritoneal inclusion cysts are surveillance, aspiration, primary surgical resection, repeat surgical resection in event of recurrence, or CRS and HIPEC [1]. The latter being the more recently advocated to prevent disease recurrence. A case study by Pradmanabhan et al. showed that their patient underwent CRS and HIPEC given concerns for PMP and intraoperatively they noticed peritoneal free-floating cysts (PFFC). The concerns with PFFC, if not treated, may result in disease recurrence by depositing elsewhere in the abdomen [1]. With complete cytoreduction they suggest it may lead to eradication of the disease process. Conversely, Rapisarda et al. suggested that the typical patient is a reproductive female, pathology is benign, and there are high rates of recurrence, therefore aspiration should be considered for symptom control as aggressive treatment may lead to infertility [8]. Given the rarity of the pathology and minimal standardized management is available, each case needs to be assessed individually and accurate diagnosis be made. Current mainstay of management includes surgical resection which can confirm histology and rule out atypia or malignancy. If a recurrence occurs, then more aggressive treatment could be considered.

## 4 Conclusion

Peritoneal inclusion cysts are a rare variant of peritoneal mesothelioma. There is a high risk of recurrence and low risk of malignant transformation. Currently first-line therapy is for surgical debulking, however, further research needs to be dedicated to this topic.

## Consent

Written informed consent was obtained from the patient for publication of this case report and accompanying images. A copy of the written consent is available for review by the Editor-in-Chief of this journal on request.

## Ethical approval

Ethical approval not applicable.

## Funding

N/A.

This research did not receive any specific grant from funding agencies in the public, commercial, or not-for-profit sectors.

## Guarantor

Dr Callie Killoran accepts full responsibility for the work and/or the conduct of the study, had access to the data, and controlled the decision to publish

## CRediT authorship contribution statement

Dr Callie Killoran – study design, writing the paper
Dr Danniel Badri – Study Design, editing the paper
Dr Alexandra Walton – data analysis/interpretation
Dr Joanna Perry-Keene– data analysis/interpretation
Dr Nicolas Copertino – study concept/design, editing the paper.

## Conflicts of interest

N/A.
